# The potential role of pumpkin seeds oil on methotrexate-induced lung toxicity

**DOI:** 10.1038/s41598-023-34143-6

**Published:** 2023-05-05

**Authors:** Aya M. Abosrea, Heba S. Aboul Ezz, Sahar M. Mahmoud, Mohamed R. Mousa, Nawal A. Ahmed

**Affiliations:** 1 Department of Zoology, Faculty of Science, Cairo University, Giza, Egypt; 2 Department of Pathology, Faculty of Veterinary Medicine, Cairo University, Giza, Egypt

**Keywords:** Biochemistry, Medical research

## Abstract

Many chemotherapeutic drugs cause adverse pulmonary reactions leading to severe pulmonary disease. Though methotrexate (MTX) is used for the treatment of cancer and other diseases, it is highly toxic with multiple adverse effects including pulmonary toxicity. Essential oils represent an open frontier for pharmaceutical sciences due to their wide range of pharmacological properties. Pumpkin seeds oil (PSO) was used to investigate its ability to alleviate methotrexate-induced lung toxicity in rats. Lung tissue from MTX-treated group revealed a decrease in malondialdehyde, glutathione, and nitric oxide accompanied by a marked inhibition in cholinesterase activity, and enhanced catalase activity, tumor necrosis factor-α, interleukin-6 and vascular endothelial growth factor levels. Analysis of PSO revealed that the oil was rich in hexadecanoic acid, decane methyl esters, squalene, polydecane, docosane, and other derivatives. Administration of PSO ameliorated the oxidant/antioxidant and proinflammatory changes induced by MTX in the lung tissue. Histological examinations confirmed the potency of PSO in reducing the histopathological alterations induced by MTX. Immunohistochemical analysis showed decreased nuclear factor-kappa B and caspase 3 expression after PSO. The present data indicated the protective efficiency of PSO against MTX-induced lung injury by decreasing oxidative damage, inflammation and apoptosis and could thus be recommended as an adjuvant therapy.

## Introduction

Chemotherapeutic agents are used extensively in solid and hematologic malignancies. Pulmonary diseases induced by chemotherapy represent particular challenges for pulmonary and critical care practitioners^1^. Many cancer chemotherapeutic drugs can cause interstitial pneumonitis/fibrosis which is the most common clinical manifestation associated with drug-induced pulmonary damage^2^.

Methotrexate (MTX) is a folic acid antagonist that competitively inhibits dihydrofolate reductase (DHFR) disrupting the conversion of dihydrofolate to tetrahydrofolate which is the primary carbon donor for purine and pyrimidine synthesis^3^. Methotrexate is used as an anti-neoplastic agent for the treatment of different types of solid organ malignancies due to its therapeutic effects. High doses of MTX were used to treat certain types of cancer but since 1990 it has been used at much lower doses to treat rheumatic diseases^4^. MTX has shown efficacy in treating several other diseases as psoriasis, psoriatic arthritis, inflammatory bowel disease, and small-vessel vasculitis^5,6^.

However, MTX was reported to be highly cytotoxic and induce multiple potential adverse effects. This limited the use of MTX due to the high incidence of associated toxicities like nephrotoxicity^7^, hepatotoxicity^8^, pulmonary toxicity^9^, cardiovascular toxicity^10^, and gastrointestinal mucositis^11^. Hypersensitivity pneumonitis is the most frequent type of pulmonary toxicity related to MTX^12^. MTX sub-acute pneumonitis is manifested by dyspnea, nonproductive cough, fever, and crackles with tachypnea on physical examination, leading to pulmonary fibrosis observed in approximately 10% of MTX Crohn disease patients^13^.

Pumpkin (*Cucurbita* spp.) from the *Cucurbitaceae* family is an annual climber plant and a traditional food known in Europe since the sixteenth century. Several studies have highlighted the protective efficiency of pumpkin seed oil (PSO) against many diseases. Its anticancer^14^, antidiabetic^15^, antioxidant^16^, antiinflammatory^17^, cytoprotective^18^, and anti-mutagenic^19^ potencies have been reported. Al-Okbi et al.^17^ reported that PSO significantly inhibited the elevated plasma tumor necrosis factor-alpha (TNF-α) and malondialdehyde (MDA) levels, thereby reducing the severity of inflammation in arthritic rat model and producing significant improvements in inflammatory and oxidative stress biomarkers.

Therefore, the present study was designed to investigate the role of PSO in alleviating the adverse effects of MTX on the levels of oxidant (malondialdehyde and nitric oxide) and antioxidant (glutathione, catalase and superoxide dismutase) parameters, and the proinflammatory cytokines (interleukin-6 and TNF-α) in the lung tissue of adult male rat. Moreover, the activity of cholinesterase enzyme and the levels of vascular endothelial growth factor responsible for angiogenesis were estimated. The analysis of PSO was performed to identify its active components. The histopathological investigation of the lung tissue in addition to the immunohistochemical examination of both nuclear factor kappa-B and caspase 3 were also carried out.

## Materials and methods

### Experimental animals

Forty adult male albino rats, weighing 160–200 g, were purchased from a local supplier. All animals were housed and kept in cages in the animal house of the Zoology Department, Faculty of Science, Cairo University, under standard conditions of light and temperature (12 h of light/dark). Rats were fed on a standard pelted chow and water was provided ad libitum. Animals were kept under observation for about 4 days for adaptation before the onset of the experiment. All experimental procedures were undertaken according to the approval of the Institutional Animal Care and Use Committee of Cairo University (CU-IACUC) No. CU/I/F/72/19**.** The study was performed in compliance with the ARRIVE guidelines. All methods were carried out in accordance with the relevant guidelines and regulations.

### Chemicals

Methotrexate was supplied by Mylan Company (France). PSO was purchased from Elhawag Company for Natural oils and Cosmetics (Egypt) licensed under no. 159, 2,150 in 2009. Glutathione, acetylthiocholine iodide, 5,5'-dithiobis-2-nitrobenzoic acid (DTNB), and phosphate buffers were obtained from Sigma Aldrich.

### Experimental design

Forty rats were used in the present experiment and were divided into four groups (ten rats in each group); control group (Cont), pumpkin seed oil (PSO) group, methotrexate (MTX) group and MTX + PSO group. Control group received a single intraperitoneal (i.p.) injection of saline solution (0.9% NaCl) followed by an oral administration of saline for 7 consecutive days. The second group was the PSO group in which rats were injected daily with pumpkin seeds oil (PSO) using an oral stomach gavage at a dose of 1 ml/kg^20^ for 7 consecutive days. The third group represented the MTX group in which animals were treated with a single i.p. injection of MTX at a dose of 20 mg/kg according to Saygin et al.^21^ followed by an oral administration of saline for 7 consecutive days. The fourth group was the MTX + PSO group in which animals were treated with a single i.p. injection of MTX (20 mg/kg) followed by PSO (1 ml/kg) for 7 consecutive days.

### Handling of tissue samples

The rats were sacrificed by sudden decapitation without anesthesia. After decapitation, the lung tissue of each animal was carefully removed, washed in ice-cold saline, blotted with filter papers, then the right half of the lung was cut on an ice-cold glass plate, and frozen for further analysis. 5 mg of each frozen lung tissue was homogenized in 5 ml 20 mM phosphate buffer (pH 7.4). The homogenates were centrifuged at 3000 r.p.m. for 10 min at 4 °C, and the obtained supernatants were stored at − 25 °C until further biochemical investigations. The other half of the lung from all treated rats was prepared for histopathology and immunohistochemical investigations.

### Methods

#### Gas chromatography mass spectrometry (GC/MS) analysis of PSO

The GC/MS analysis of PSO was performed using a Thermo Scientific, Trace GC Ultra/ISQ Single Quadrupole MS, TG-5MS fused silica capillary column (30 m, 0.251 mm, 0.1 mm film thickness). For GC/MS detection, an electron ionization system with ionization energy of 70 eV was used. Helium was used as the carrier gas at a constant flow rate of 1 ml/min. The injector and MS transfer line temperature was set at 280 °C. The oven temperature was programmed at an initial temperature of 40 °C (hold 3 min) to 280 °C as a final temperature at an increasing rate of 5 °C/min (hold 5 min).

The quantification of all the identified components was investigated using a percent relative peak area. A tentative identification of the compounds was performed based on the comparison of their relative retention times and mass spectra with those of the NIST, WILLY 9 library data of the GC/MS system.

### Biochemical analysis

#### Determination of malondialdehyde (MDA) content

Malondialdhyde (MDA; the end product of lipid peroxidation) was determined in rat lung tissue homogenates according to the method of Ohkawa et al.^22^ using kit No. MD 2529 (Biodiagnostic, Egypt). The reaction of thiobarbituric acid (TBA) with MDA takes place in an acidic medium at a temperature of 95 °C for 30 min to form thiobarbituric acid reactive substances (TBARS). The absorbance of the samples and standard was read against blank at 534 nm in a Helios Alpha Thermospectronic (UVA 111615, England) spectrophotometer. Malondialdhyde content in the lung tissue homogenate (sample) was measured in nmol/g tissue.

#### Determination of glutathione (GSH) content

The concentration of GSH was measured using Kit No. GR 2511 (Bio diagnostic, Egypt) which is based on the method described by Beutler et al.^23^. This method is based on the reduction of 5,5' dithiobis (2-nitrobenzoic acid) (DTNB) with glutathione to produce a yellow compound. The reduced chromogen was directly proportional to reduced glutathione concentration and its absorbance was measured spectrophotometrically at 405 nm. GSH concentration was expressed in mmol/g tissue.

#### Determination of nitric oxide content

Nitric Oxide (NO) is synthesized in the biological system by the enzyme nitric oxide synthase (NOS).The final products of NO in vivo are nitrite (NO^2-^) and nitrate (NO^3-^). Determination of NO content was carried out according to the method of Montgomery and Dymock^24^ which depends on the addition of Griess reagent in acidic medium. Griess reagent converts nitrite into a deep purple azo compound. The formed nitrous acid diazotizes sulphanilamide and the product is coupled with N-(1-naphthyl) ethylene-diamine. Absorbance of the formed reddish-purple azo dye was measured spectrophotometrically at 540 nm in a Helios Alpha Thermospectronic (UVA 111615, England) spectrophotometer.

#### Determination of catalase (CAT) activity

Catalase activity in rat lung tissue homogenate was determined by the method of Aebi^25^ using Kit No. CA 2517 (Biodiagnostic, Egypt). The method is based on the reaction of CAT with a known quantity of H_2_O_2_, as each unit of CAT decomposes 1 µM of H_2_O_2_ per min at 25 °C, at pH 7.0. The reaction is stopped after exactly 1 min with catalase inhibitor. In the presence of horse radish peroxidase (HRP), the remaining H2O2 reacts with 3,5 dichloro-2-hydroxybenzene sulfonic acid (DHBS) and 4-amino-phenazone (AAP) to form a quinone imine dye whose color intensity is inversely proportional to the amount of catalase in the sample. The decomposition of H_2_O_2_ was followed directly by the decrease in absorbance at 240 nm. The difference in the absorbance per unit time is a measure of the CAT activity.

#### Determination of superoxide dismutase (SOD) activity

Superoxide dismutase (SOD) activity in lung tissue homogenate was assayed according to the procedure of Nishikimi et al.^26^. The assay depends on the ability of SOD to inhibit phenazine methosulphate-mediated reaction of nitrobluetetrazolium dye. SOD catalyzes the dismutation of the superoxide anion to molecular oxygen and hydrogen peroxide. The increase in absorbance was measured at 560 nm for 5 min at 25 °C for blank and sample.

#### Determination of cholinesterase (ChE) activity

The present study used a modification of the method of Ellman et al.^27^ as described by Gorun et al.^28^. The method measures the rate of production of thiocholine as acetylthiocholine hydrolysis proceeds. ChE is incubated with acetylthiocholine for a specific time interval and the reaction is then stopped with a reagent containing the color indicator DTNB. The color was read immediately at 412 nm and ChE activity was determined as µmol SH/g tissue/min (SH sulfhydryl group).

#### Determination of inflammatory cytokines

##### Tumor necrosis factor alpha (TNF- α)

Tumor necrosis factor-alpha (TNF-α) was measured using rat TNF-α Elisa kit Catalog No. SG-20127 which was obtained from SinoGeneClon Biotech Co., Ltd. (Hangzhou, China). The developed color was read at 450 nm using a microtiter plate reader. The concentration was then calculated from a standard curve.

##### Interleukin 6 (IL-6)

Interleukin-6 (IL-6) was measured using rat interleukin-6 Elisa kit Catalog No. SG-2026 which was supplied by SinoGeneClon Biotech Co., Ltd. (Hangzhou, China). The color change was measured at a wavelength of 450 nm using a microtiter plate reader. The concentration of IL-6 in the samples was then determined from a standard curve.

#### Determination of vascular endothelial growth factor (VEGF)

Vascular endothelial cell growth factor (VEGF) was measured using rat vascular endothelial growth factor Elisa kit Catalog No. SG-20402 which was purchased from SinoGeneClon Biotech Co., Ltd. (Hangzhou, China). The color change was measured at wavelength of 450 nm. The concentration was then calculated by comparing the optical density of the samples to the standard curve.

### Histopathology

Lung sections were collected from different groups and preserved in 10% neutral buffered formalin for histopathological and immunohistochemical evaluation. After alcohol dehydration and paraffin embedding steps, tissues were cut at 5 µm thickness on glass slides. Hematoxylin and eosin stains (H&E) were used to stain the tissue sections. The lung sections were examined using bright field microscope (Olympus BX43) and digital images were captured using fixed digital camera (Olympus DP27). The lung score system was performed as previously mentioned^29^.

### Immunohistochemical analyses

Lung Sects. (5 μm) were cut from paraffin blocks into positive charged glass slides. Ordinary steps of deparaffinization and rehydration were performed via a series of graded alcohol. Heat-induced epitope retrieval was conducted. The lung sections were washed then protein blocking using bovine serum albumin (BSA) and hydrogen peroxide was applied. After that, primary antibodies (mouse monoclonal Anti-NF-κB p65 (sc-8008) were incubated with caspase-3 (sc-65497, Santa Cruz Biotechnology Inc., Dallas, TX, USA) at a dilution of 1:150 overnight at 4 °C. After that, tissue sections were incubated with horseradish-peroxidase (HRP) labeled goat anti-mouse secondary antibody (Abcam, Cambridge, UK) for 2 h then 3,3′-diaminobenzidine (DAB) Substrate detection kit (Thermo Scientific) was applied. Counterstaining was done using Mayer's hematoxylin, dehydration followed by xylene clearance and mounting in dibutylphthalate polystyrene xylene (DPX). Area % of positive reaction was quantified using digital software (Cell Sens dimensions, Olympus software). Negative control step was achieved via omitting the primary antibody incubation step.

### Statistical analysis

All data were expressed as mean ± standard error of mean (S.E.M.). Significance was determined at p < 0.05. Comparisons between the means of different groups of animals and those of control animals were carried out using one-way analysis of variance (ANOVA) for each time interval. All statistical analyses were done using SPSS (Statistical Package for Social Sciences, version 14). When statistically significant, ANOVA test was followed by Duncan as post hoc test for multiple comparisons.

### Ethics approval and consent to participate

All experimental procedures were undertaken according to the approval of the Institutional Animal Care and Use Committee of Cairo University (IACUC) No. CU/I/F/72/19.

## Results

### Identification of pumpkin seed oil (PSO) components using GC/MS

**Figure 1:**
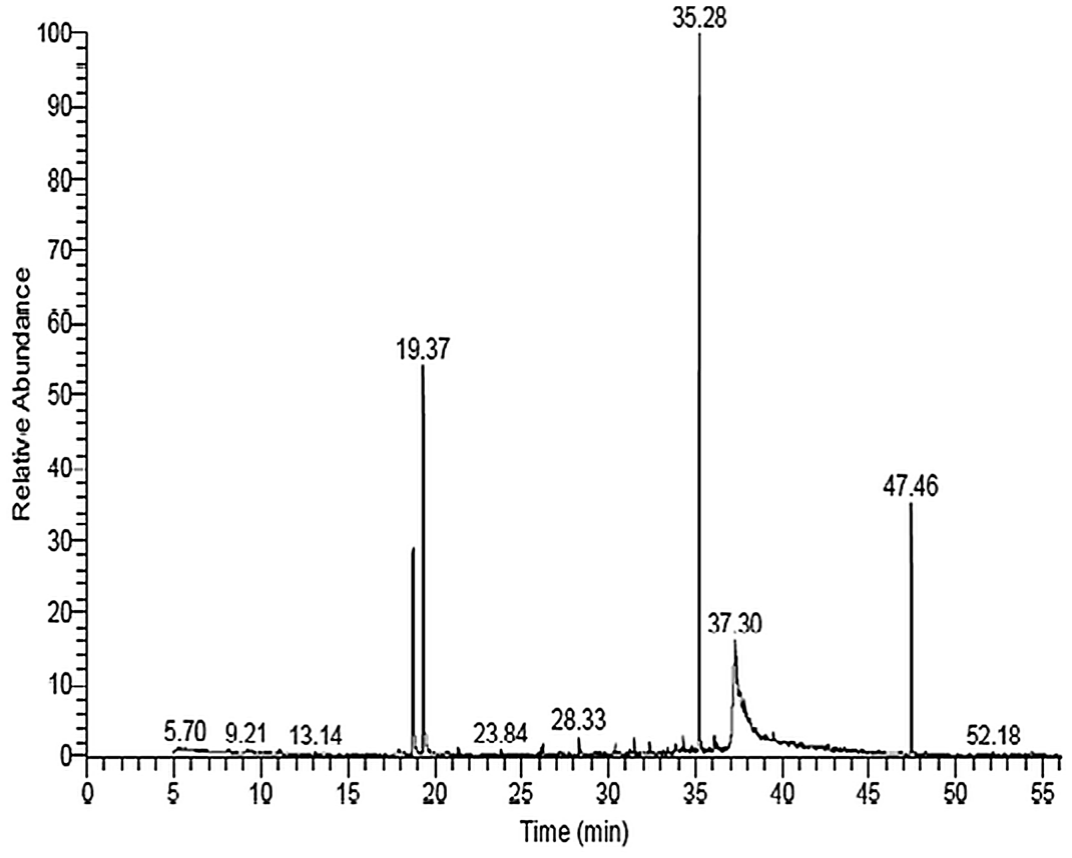
The Chromatogram and Spectrum of Identified Compounds From Pumpkin Seed Oil (PSO) Using Trace GC Ultra / ISQ Single Quadrupole MS, TG- 5MS Fused Silica Capillary Column.

Identification of pumpkin seed oil (PSO) components was performed based on the comparison of their relative retention time and mass spectra with those of the NIST, Willy 9 library data of the GC/MS system. The spectrum of the unknown components was compared with the spectrum of the known components stored in the Willy 9 library. The retention time, name, molecular weight and structure of the components of the test materials were ascertained. The GC/MS analysis of PSO has shown its phytochemical constituents and their peaks (Fig. 1), which contribute to the medicinal activity of the plant. The identification of the active components of PSO with their retention time and percentage composition revealed that PSO used in this study was rich in hexadecanoic acid (42.06%), decane methyl esters (25.9%), squalene (11.16%), polydecane (5.56%), docosane (2.56%), and other derivatives (Table 1).

**Table 1:** Identification of Pumpkin Seed Oil (PSO) Components Using GC/MS.

Compound name	RT/min	Area %	MW	MF
1. N, N Dimethyl vinyl amine	5.14	0.28	71	C4H9N
2. 3 (Prop2enoyloxy) tetra decane	9.21	0.31	268	C17H32O2
3. OCimene	11.06	0.27	134	C10H14
4. Methyl 3 Methyl 4 ( trimethysilyl) 2butenoate	13.61	0.20	186	C9H18O2Si
5. 2, 4 Decadien-1-ol	17.96	0.35	154	C10H18O
6. 2,4Decadienal	19.37	15.85	152	C10H16O
7. Hentriacontane	21.41	0.22	436	C31H64
8. Docosane	23.85	0.32	310	C22H46
9. 2,3 Dihydro5,10,15,20te traphenyl22 H, 24Hprophyrin	25.09	0.19	616	C44H32N4
10. Pentadecane	26.15	0.33	212	C15H32
11. Farnesol	26.26	0.51	222	C15H26O
12. 3,4,5,6Tetrakis(pchlorophenoxy) 1,2dicyanobenzene	27.22	0.22	632	C32H16Cl4N2O4
13. Pentadecane	28.33	0.83	212	C15H32
14. Methyl DELTA.meso(Methylthio)methyl)mesopyropheophorbide A	28.46	0.27	671	C36H40CuN4O3S
15. MethylE7hexadecene	29.01	0.19	238	C17H34
16. Heptadecane,2,6,10,14tetramethyl	30.41	0.41	296	C21H44
17. Dodecanal	31.25	0.30	184	C12H24O
18. Tetradecanoic acid, Trimethylsilyl ester	31.50	0.76	300	C17H36O2Si
19. Pentadecane	32.40	0.52	296	C21H44
20. Decanoic acid, methyl Ester	33.02	0.23	186	C11H22O2
21. 1-Hexacosanol	33.19	0.34	140	C10H20
22. 1-H. Indole2carboxylic acid,1(trimethylsilyl)5[(trimethylsilyl)oxy],trimethylsilyl ester	33.41	0.30	393	C18H31NO3Si3
23. n-Hexadecanoic Acid	33.90	0.75	256	C16H32O2
24. Dodecane, 2,6,10trimethyl	34.29	0.75	212	C15H32
25. Hexadecanoic acid, trimethylsilyl ester	35.27	42.06	328	C19H40O2Si
26. Docosane	35.61	0.27	310	C22H46
27. Isochiapin B	35.88	0.76	346	C19H22O6
28. Octadecane	36.12	1.03	254	C18H38
29. 9-Octadecenoic acid, methyl ester	36.25	0.54	296	C19H36O2
30. Hexadecen,1-ol, 3,7,11,15tetramethyl, [R[ R*,R*(E)]]	36.35	0.21	296	C20H40O
31. Docosane	36.78	0.19	490	C35H70
32. 13, Oxabicyclo[10.1.0-Tridecane]	37.30	6.73	182	C12H22O
33. 9-Octadecenoic acid (Z) hexadecyl ester	37.74	0.21	506	C34H66O2
34. Docosane	37.85	0.54	310	C22H46
35. Linoleic acid ethyl ester	38.18	0.25	308	C20H36O2
36. 7, Pentatriacontene	38.48	0.29	490	C35H70
37. 31, dodecaene	39.18	0.29	662	C39H42N4O6
38. Docosane	39.51	0.60	310	C22H46
39. Octadecane	39.63	0.24	296	C20H40O
40. Dodecachloro3,4benzo-phenanthren	40.63	0.24	636	C18Cl12
41. Docosane	41.10	0.19	310	C22H46
42. Docosane	41.67	0.25	310	C22H46
43-Docosane	42.64	0.20	310	C22H46
44. Octan2one, 3,6dimethy	43.18	0.24	156	C10H20O
45. Octadecane	43.58	0.26	254	C18H38
46. Squalene	47.46	11.16	410	C30H50
47. Cholesta8,24dien3ol,4methy	54.40	0.30	398	C28H46O

### Biochemical results

#### The oxidant/antioxidant parameters

##### Malondialdehyde (MDA) levels

**Figure 2:**
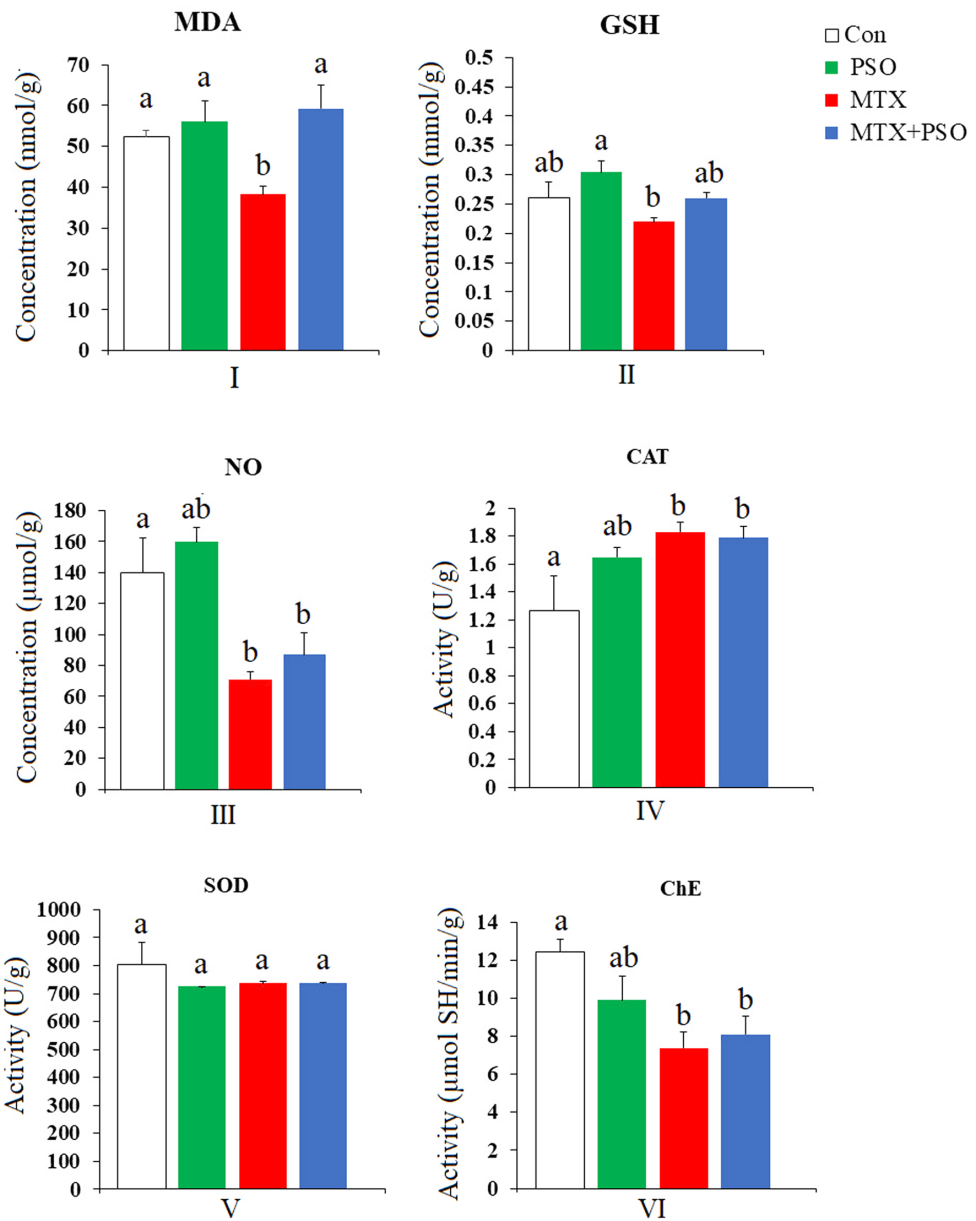
The effect of oral administration of pumpkin seed oil (PSO) on malondialdehyde (MDA) (I), glutathione (GSH) (II), nitric oxide (NO) (III) levels and catalase (CAT) (IV), superoxide dismutase (SOD) (V), and cholinesterase (ChE) (VI) activities in methotroxate-intoxicated rat lungs. Different letters indicate significantly different means (p value ˂ 0.05). Same letters indicate non significant changes.

A single i.p. injection of MTX caused a marked decrease in MDA levels in the lung of adult male rats after eight days of treatment (Fig. 2). The observed decrease in MDA level was significant at p value ˂ 0.05 compared to the control group, recording a percentage difference of − 26.72%. The oral administration of PSO for eight consecutive days induced a slight non significant increase in MDA levels in the lungs of rats as compared to control group. Treatment of MTX-injected rats with PSO prevented the change in the MDA level.

##### Glutathione (GSH) level

The intraperitoneal injection of MTX provoked a non significant decrease in GSH levels in the lungs of MTX-treated rats as compared to control group and MTX group treated with PSO (Fig. 2). However, the daily oral administration of PSO induced a non significant increase in GSH content in the lung tissue of MTX + PSO-treated rats as compared to control value.

##### Nitric Oxide (NO) level

The data represented in Fig. 2 revealed that a single MTX injection to adult male rats induced a marked decrease in NO levels in the lung recording − 49.13% below the control levels. The oral administration of PSO slightly improved NO levels from − 49.13 to − 37.68% in rat lung tissue compared to control values.

##### Catalase (CAT) activity

The present data demonstrated that rats injected with MTX showed a significant increase in CAT activity, being 44.3% above the control values. Treatment of MTX-intoxicated rats with PSO induced an increase in CAT activity with a percentage difference of 41.1% as compared to control group (Fig. 2).

##### Superoxide dismutase (SOD) activity

Analysis of data concerning the activity of SOD in lung tissue from the investigated groups yielded non significant changes with a slight decrease in the enzyme activity after PSO and MTX treatments alone or combined with each other (Fig. 2).

##### Cholinesterase (ChE) activity

A single MTX injection was found to induce a marked inhibition in cholinesterase enzymatic activity recording 40.5% below the control values (Fig. 2). When MTX-intoxicated rats were treated daily with PSO for eight days, an improvement in the enzyme activity was obtained, being − 34.8% but still significantly lower than control group.

### Pro-inflammatory cytokines

#### Tumor necrosis factor-alpha (TNF-α) Level:

**Figure 3:**
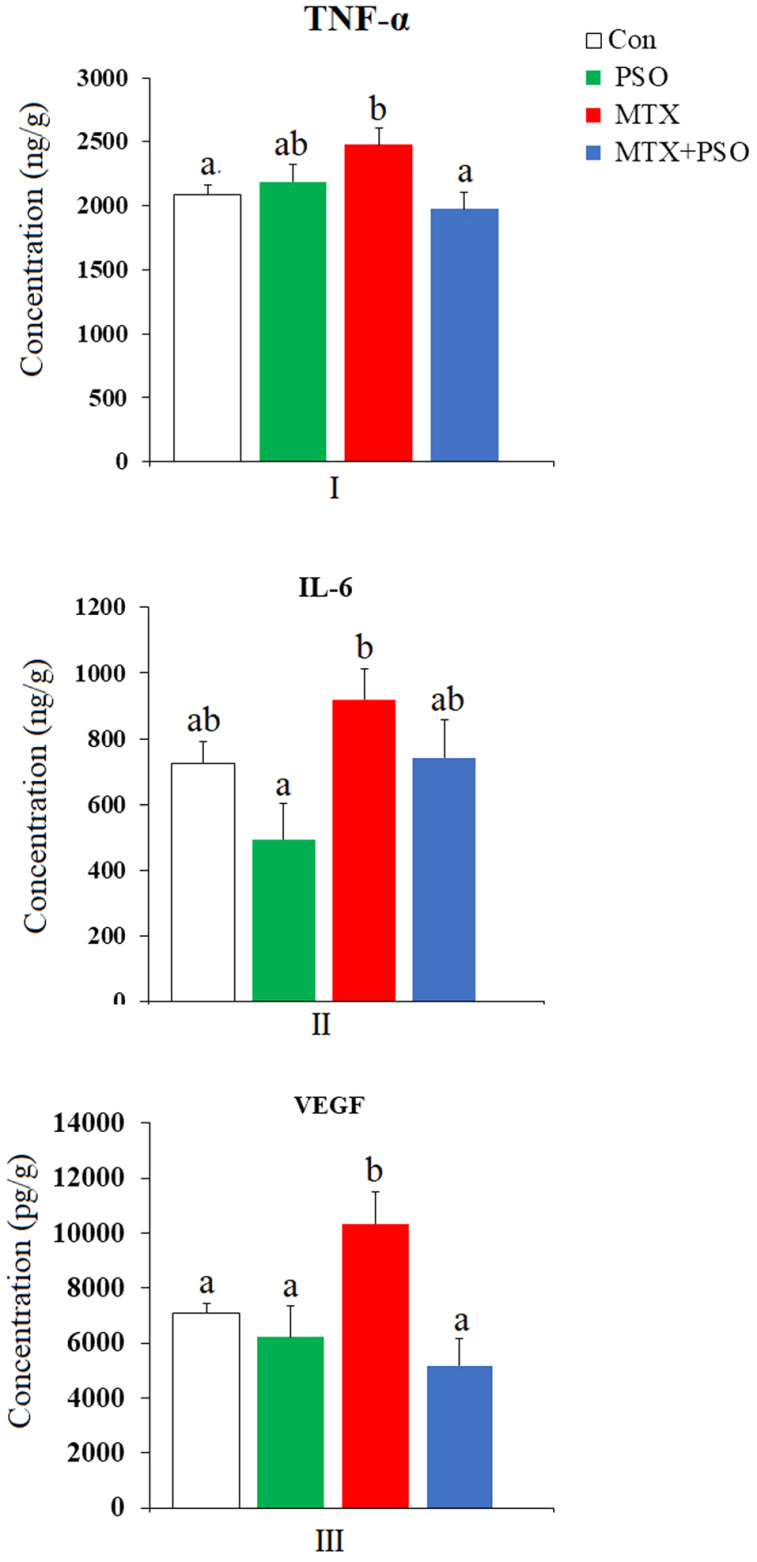
The effect of oral administration of pumpkin seed oil (PSO) on tumor necrosis factor-alpha (TNF-α) (I), interleukin-6 (IL-6) (II) and vascular endothelial growth factor (VEGF) (III) levels in methotrexate-intoxicated rat lungs. Different letters indicate significantly different means (p value ˂ 0.05). Same letters indicate non significant changes.

A single i.p. injection of MTX induced a significant elevation in TNF-α level in rats’ lungs recording a percentage difference of 19.10% versus control values (Fig. 3). Treatment of MTX-intoxicated rats daily with PSO for eight days ameliorated the increase in TNF-α in lung tissue lowering the percentage difference to − 5.11% compared to the control group.

#### Interleukin-6 (IL-6) level

The levels of interleukin-6 (IL-6) in lung tissue of PSO-treated rats showed a non significant decrease (− 31.77%) compared to control group (Fig. 3). On the contrary, MTX injection evoked a non significant increase in IL-6 recording 26.93% above the control group. The daily oral administration of PSO to MTX-injected rats for eight days attenuated the increase in IL-6 resulting from MTX injection reducing the percentage difference from 26.93 to 2.32%.

#### Vascular endothelial growth factor (VEGF) Level

A marked increase in VEGF levels was recorded in lung tissue of MTX-injected rats (Fig. 3). The observed increase was significant at p value ˂ 0.05 versus the control and PSO groups and recorded 45.98% compared to the control values. Treatment of MTX-injected rats with PSO induced a non significant decrease in VEGF levels after 8 days of treatment, the percentage difference being reduced from 45.98% after MTX to − 26.89% after PSO.

### Histopathological and immunohistochemical examinations

#### Histopathology

**Figure 4:**
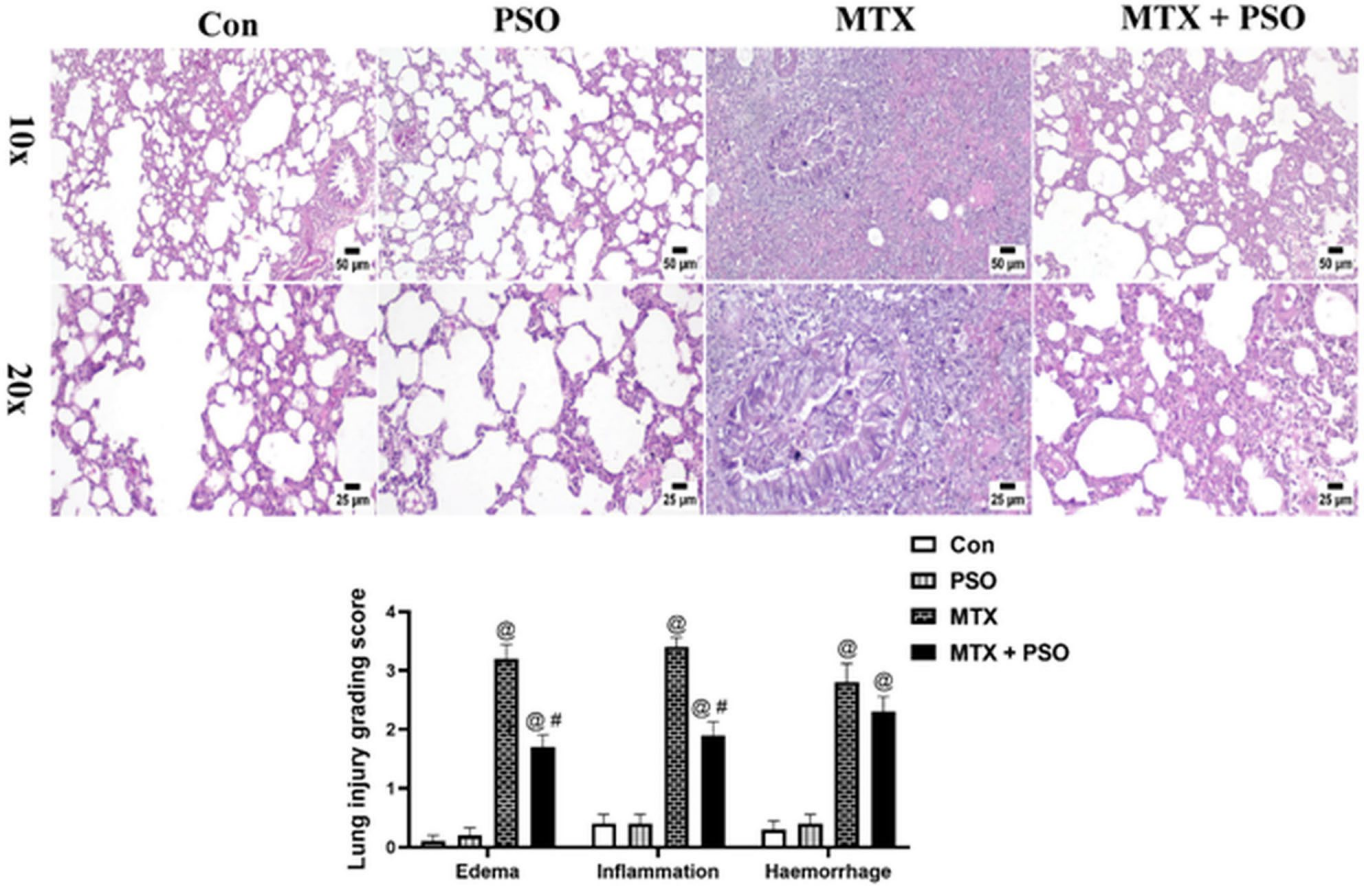
Effect of PSO on recovery of lung tissue from MTX-induced lung injury (H&E). Control and PSO groups showed normal histological structure of lung alveoli, bronchi and interstitial tissue. MTX group showed severe interstitial pneumonia with abundant edema and heavy mononuclear inflammatory cells infiltration, the higher magnification (20x) showed that the bronchiolar lumen was occluded with mucous exudate and inflammatory cells with desquamated epithelial cells. MTX + PSO group showed marked reduction of inflammatory reaction in the lung parenchyma. Chart represents the lung score injury of different groups. Values are expressed as means ± SE. @ significant from Con, # significant from MTX. Significant difference is considered at p < 0.05.

Lung sections from control and PSO groups showed normal histological structure of lung tissue without any detectable alterations. Adversely, examination of MTX group revealed severe lung injury. The interstitial tissue was markedly expanded with numerous mononuclear inflammatory cells infiltration associated with abundant edema and variable hemorrhagic areas. The bronchi and bronchioles displayed mucus exudates in their lumens mixed with sloughed epithelial cells, inflammatory cells and tissue debris. Administration of PSO to MTX-intoxicated rats induced marked protection of the lung tissue that was characterized by fewer numbers of inflammatory cells infiltration, mild edema and hemorrhages occupying the lung alveoli and inter-alveolar septa. The lung scoring system revealed a significant increase in MTX group when compared to control or PSO groups in all evaluated parameters. However, MTX + PSO group showed a significant decrease in comparison with MTX group concerning edema and inflammation (Fig. 4).

#### Immunohistochemistry

**Figure 5:**
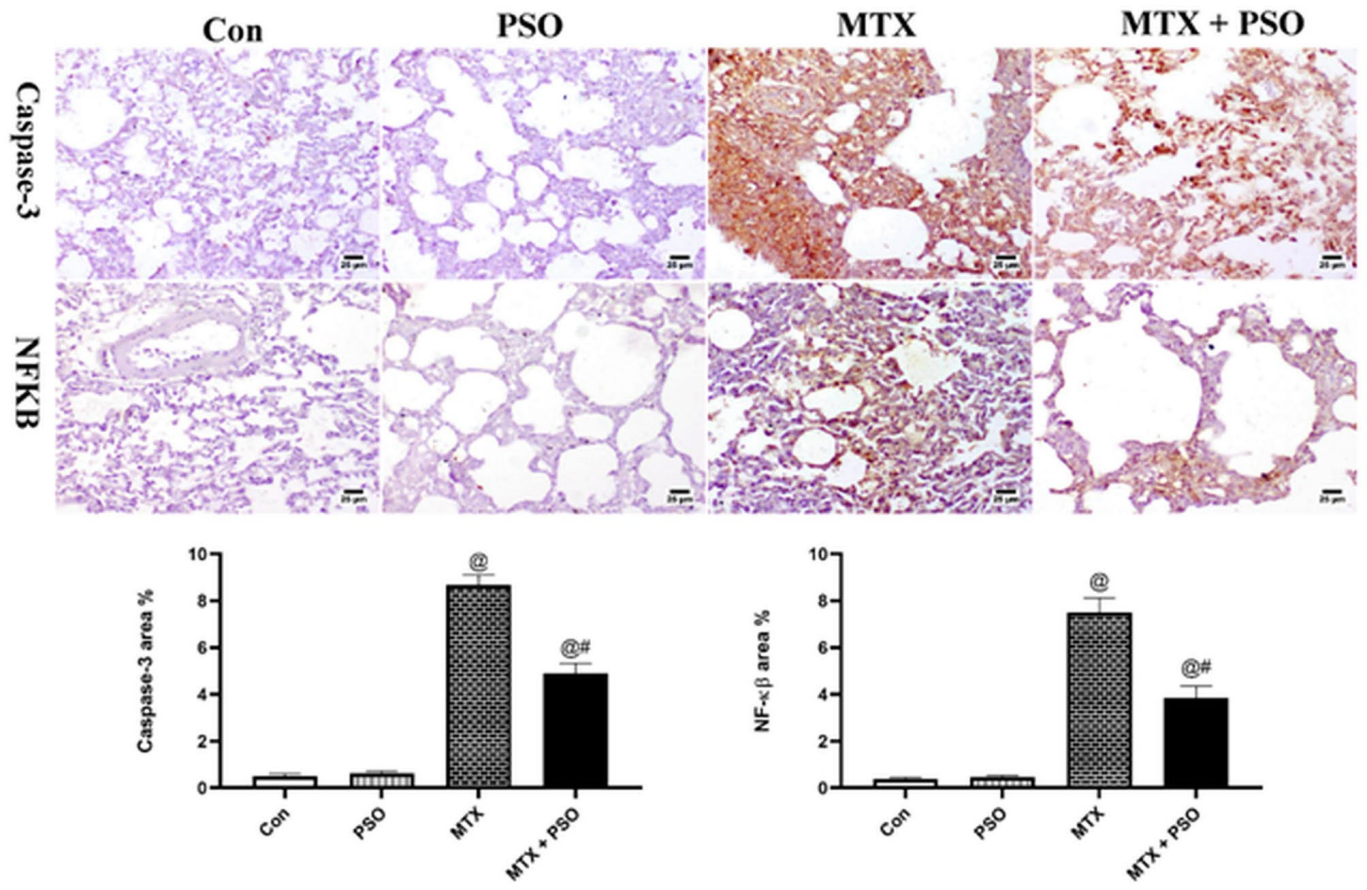
Immunohistochemical staining of caspase-3 and NF-kβ in different groups. Control and PSO groups showed limited to negative expression in both markers. MTX showed strong positive expression in the inflamed lung sections concerning caspase-3 and NF-kβ. Remarkable decrease in immune staining is detected in MTX + PSO group of both caspase-3 and NF-kβ. Charts represent area % expression of immune markers in different groups. Values are expressed as means ± SE. @ significant from Con, # significant from MTX. Significant difference is considered at p < 0.05.

Evaluation of caspase-3 and NF-kβ was performed in lung sections of different groups. Concerning caspase-3 immune staining, control and PSO showed weak to negative expression in the alveolar lining and in the interstitial tissue. However, strong positive expression of caspase-3 was detected in the interstitial tissue, alveoli and lining epithelium of bronchi and bronchioles of MTX group. Moderate expression was detected in MTX + PSO group. Similar results were obtained in NF-kβ immune staining. The statistical analysis of area % expression showed a significant increase in MTX group when compared to other groups regarding caspase-3 and NF-kβ staining (Fig. 5).

## Discussion

Lung damage may result from drug-induced pulmonary toxicity or autoimmune-mediated inflammation^12^. The therapeutic use of MTX is limited by its toxicity^21^ which leads to alteration of the dosage regimen, or complete drug withdrawal^12^.

The increased levels of inflammatory markers, TNF-α, IL-6 and VEGF, in the present study, clearly demonstrate the cytotoxic pro-inflammatory effects of MTX on lung tissue. The increase in pro-inflammatory cytokines may be explained by the ability of MTX to up regulate the expression and secretion of these cytokines which may further enhance the production of ROS and the subsequent lung tissue damage.

Compared to other organs, lungs are highly vulnerable as they are directly exposed to high levels of atmospheric oxygen. Hence, the respiratory epithelium is a major target for oxidative stress. Therefore, the enzymatic and non-enzymatic antioxidant defense systems in the lung are rich and highly efficient to protect it from oxidant-induced damage^30^.

The present study revealed that a single MTX injection caused a significant decrease in the content of the oxidative markers MDA and NO, and a nonsignificant decrease in GSH levels and SOD activity in rat lungs. However, an elevation in CAT activity was recorded in the lung tissue of MTX-injected rats.

Oxidative stress induces radical mediated damage to cellular bio-membranes causing lipid peroxidation, which leads to the generation of oxidized products capable of modifying DNA, protein, and other macromolecules^31^. Reactive oxygen species (ROS) target the major cellular macromolecules leading to their damage and subsequently cell death in response to oxidative insult. This triggers the onset and progression of tissue damage and oxygen may increase active free radical exposure^32^. The present inflammation induced by MTX suggests that the production of ROS may be in progress. The increase in CAT enzyme supports this notion.

The histopathological examination results of the present study indicated severe lung injury in the MTX-treated group, which revealed marked expanded interstitial tissue with numerous mononuclear inflammatory cells, infiltration, abundant edema and variable hemorrhagic areas with mucus exudates in the lumen of bronchi and bronchioles mixed with sloughed epithelial cells, inflammatory cells and tissue debris. Similarly, it has been shown that MTX–induced pulmonary toxicity was correlated with hypersensitivity pneumonitis^33^ characterized by interstitial lymphocyte infiltration with hyperplasia, formation of small granulomatous areas, and sometimes eosinophilic infiltration, other patterns showing obstructive pneumonitis, acute interstitial pneumonia, pulmonary fibrosis, and pleural effusion^34^.

The present findings do not agree with the reported increase in MDA level and decrease in CAT and SOD activities in rat lung after MTX^35^. This may be explained by the short time interval applied in the present study. Hence, the activity of SOD and the level of GSH were not changed as compared to the control. However, the present increase in CAT activity after MTX suggests that this enzyme is the first antioxidant to respond to the generation of free radicals in lung tissue.

The histopathological alterations observed in the present study indicate severe lung injury resulting from MTX. The progression of tissue damage will eventually lead to the decrease in MDA levels due to the cellular death. Several histopathological studies revealed that MTX-induced lung injury caused significant lymphocytic inflammation and congestion with pneumocyte hyperplasia, epithelial degeneration and fibrous tissue deposition^36^. Injury of the lung epithelial cells which line the airspaces increases mucus secretion, enhances neutrophils influx into the lungs, and activates transcription factors and gene expression of pro-inflammatory mediators^37^. This explains the presence of mucus exudates in the lumen of bronchi and bronchioles among various cells and tissue debris in the lung sections of MTX-treated rats.

Catalase catalyzes the dismutation of hydrogen peroxide to water and oxygen^38^. In the lung, CAT is localized in alveolar type II pneumocytes and macrophages^39^. It is considered to be the most important antioxidant enzyme consuming exogenous hydrogen peroxide in type II pneumocytes, which are the most resistant cell types in the lung^40^. The significant increase in CAT activity, in the present study, in lung tissue after MTX injection emphasizes the importance of catalase as a major antioxidant that confers protection to the lung under conditions of increased oxidative stress.

There are three isoforms of NOS in the human respiratory system. Neuronal NOS (nNOS) was detected in nonadrenergic non-cholinergic nervous system, airway nerves and epithelium. Endothelial NOS (eNOS) is present mainly in endothelial cells of the lung vasculature while inducive NOS (iNOS) has been associated with the pro- and anti-inflammatory responses. All isoforms transform the L-arginine into L-citrulline and release nitric oxide^41^. Any change of their activity may lead to alteration in NO level and contribute to the pathogenesis of various respiratory diseases^42^.

It has been reported that eNOS is activated by TNF-α and other inflammatory stimuli^43^. It may be suggested that the activation of eNOS by the elevated inflammatory cytokines induced by MTX may lead to the generation of NO which interacts with superoxide radical producing peroxynitrite^44^. This is a strong oxidant and can precipitate cellular damage either directly by nitration of tyrosine leading to irreversible dysfunction of important proteins, or indirectly by initiating the production of other reactive molecules with cellular toxicity^42,45^. NO was reported to exert vasoprotective effects on the endothelium keeping blood vessels dilated, and also controlling blood pressure^46^.The reduction in NO may interfere with its vasoprotective effects thus promoting MTX toxicity in the lung tissue.

Several studies pointed at the promising therapeutic effects of PSO^14–19^. The present results revealed that the ability of PSO to attenuate MTX-induced biochemical changes in rat lung was evident after PSO administration for 8 consecutive days. PSO maintained MDA and GSH near control levels and slightly improved NO levels in rat lung tissue compared to control values. Treatment of MTX-injected rats with PSO also maintained the increased activity of CAT in the lung tissue.

Analysis of PSO using GC/MS, in the present study, showed its richness in hexadecanoic acid, decane methyl esters, squalene, polydecane, docosane, and other derivatives. Hexadecanoic and octadecanoic acids were documented as antioxidant^47^, anticancer^48^, antiapoptotic^49^ and anti-inflammatory^47^ agents. The present results concerning analysis of the active constituents of PSO were in line with other studies reporting high amounts of free fatty acids (oleic, linoleic, palmitic, and stearic acids) in pumpkin oil^50,51^. Furthermore, results of the present study also agree with Omar and Sarhan^52^ who attributed the improvement in acid-aspiration pneumonia model after pumpkin seed oil treatment to its composition.

Squalene is a lipophilic isoprenoidtriterpene that contributes to the antioxidant effect of pumpkin oil as a guard against membrane lipid peroxidation^53^. Squalene oxidation was reported to occur earlier than other lipids, blocking the peroxidative reactions as it breaks lipid peroxidation chains thus stabilizing and protecting cell membranes^54^. Moreover, emerging evidence supported the involvement of squalene in the regulation of glutathione peroxidase, CAT, SOD and glutathione S-transferase expression and activation, prevention of SOD and CAT alterations and reconstitution of GSH^55^.

In addition, PSO was reported to decrease GST activity that catalyzes GSH conjugation and GR activity but elevated GSH level indicating lower turnover of GSH, an essential regulator of the cellular redox state^56^. The present elevated CAT activity reflected increased concentrations of hydrogen peroxide in rats’ lung tissue exposed to MTX, and the persistence of PSO in reducing the high influx of hydrogen peroxide.

It can be suggested that the ability of PSO to maintain the increased levels of CAT induced by MTX, in the present study, was due to the potent antioxidant constituents of PSO. The elevated CAT activity may indicate increased production of free radicals as hydrogen peroxide and supports the role of CAT as one of the important antioxidants in lung tissue. Several reports confirmed the potency of different constituents of PSO. Eraslan et al.^57^ suggested that pumpkin seed oil may have caused physiological alterations in the activities of lung SOD, brain CAT and liver GSH-Px after subacute aflatoxin poisoning in mice due to its potential of eliminating free radicals generated under normal biological conditions. Similarly, it has also been reported that pumpkin seed oil/extract/isolate alters the same antioxidant enzyme activities^58^. Recently, it has been reported that treatment of diabetic mice with 2% squalene increased the activity of liver catalase and glutathione peroxidase, while a dose of 600 mg of squalene increased catalase and superoxide dismutase activities and reduced hydrogen peroxide levels^59^.

In line with the present findings, Shaban et al.^60^ suggested that the antioxidant activities of pomegranate extracts could be related to their constituents, including phenolic components, fatty acids (such as punicic acid and conjugated α-linolenic acids), phytosterols, and triterpenoids. Moreover, Habashy et al.^61^ found that the improvement of the antioxidant defense system by Vitis vinifera polyphenols fraction (VVPF) in the lung tissue was higher than in other studied organs and suggested that this may be due to the potent reducing power of VVPF and its ability in quenching ABTS radical. This potency was related to the phenolic content of VVPF, including vanillic, gallic, caffeic, p-coumaric, syringic, ferulic, salicylic, and ellagic acids, along with the flavonoids and resveratrol and its efficiency in scavenging peroxide, superoxide, and hydroxide radicals^62^.

Omar and Sarhan^52^ found that pumpkin oil partially attenuated the histological and ultra structural alterations and reduced inducible NO synthase immune-expression in lung tissue of experimentally-induced acid aspiration pneumonia model.

Thus, the failure of PSO to restore the present decrease in NO levels may be due to the reduced iNOS expression which affects NO production in the lung tissue of MTX-treated rats. This may be beneficial in reducing the nitrosative stress and peroxynitrite formation in these animals.

In the present study, MTX injection resulted in a marked reduction in cholinesterase (ChE) activity in the lung tissue. Besides its classical role in hydrolyzing acetylcholine (ACh) in the central and peripheral nervous systems, acetylcholinesterase (AChE) was also associated with stress responses and inflammation^63^. Abnormal expression and structural alterations of AChE were observed in different tumors, as reduced activity may contribute to lung cancer growth^64^. On the other hand, AChE was found to be involved in tumor suppression by its participation in apoptosis indicating its potential role as a marker and regulator of apoptosis and tumor development^65^.

The present reduction in ChE activity in the lung tissue of MTX-intoxicated rats may be due to the state of inflammation and subsequent ROS generation that developed in the lung tissue. The study of Tsakiris et al.^66^ revealed that AChE is very sensitive to free radicals and that hydroxyl radicals participate in AChE inhibition. The decrease in the enzyme activity may also be related to the mechanism of action of MTX due to the involvement of the enzyme in tumourgenesis and proliferation.

Pioneer reports highlighted the potential role of oleic acid in the cholinergic system^67^, and showed a choline acetyltransferase (ChAT)-like activity responsible for increased production of ACh by isolated rat brain synaptosomes bound to neuronal plasma membranes. In addition, it has been reported that oleic acid enhanced choline uptake^68^ and affected the expression of ChAT, which probably promotes ACh production^69^. Furthermore, Abed et al.^70^ reported that squalene and palmitic acid exhibited different rates of inhibition against AChE.

The inability of PSO to restore ChE activity in MTX-intoxicated may be explained by two mechanisms. First, PSO constituents enhance choline uptake and choline acetyltransferase activity and inhibit ChE activity and hence promote cholinergic transmission. Second, the decrease in ChE may be a compensatory mechanism that aims to increase ACh since neuronal or non-neuronal sources of ACh could decrease inflammation by their local action on macrophages, through the alpha-7 nicotinic ACh receptor thus reducing nuclear translocation of the transcription factor NF-κB, and macrophage production of the pro-inflammatory cytokine TNF-α^71^.

Increased levels of TNF-alpha and IL-6, in the present study, indicate that MTX has pro-inflammatory effects on lung tissue.

TNF-alpha is a potent pro-inflammatory cytokine responsible for the pathogenesis of chronic inflammatory diseases and oxidative stress. Its actions are achieved by regulating growth, proliferation, differentiation, and viability of activated leukocytes, inducing cellular release of other cytokines and chemokines^72^ and ensuring the death of damaged cells by apoptosis^17^. The binding of TNF-α to its receptor triggered and propagated the signaling cascades leading to the development of local or systemic inflammation which activates NF-κB forming a positive feedback mechanism which enhanced the inflammatory process^17,72^.

IL-6 is a pleiotropic cytokine having both pro- and anti-inflammatory properties. It is produced by different immune cells and synergizes with TNF-alpha and IL-1 to promote systemic inflammatory response^73^.

Studies documented increased TNF-alpha, interleukin-1 (IL-1), interleukin-8 (IL-8), and monocyte chemotactic protein-1 in acute MTX-induced pulmonary toxicity^74,75^. Kurt et al.^76^ attributed the high levels of TNF-alpha, MDA, myeloperoxidase (a component of the antioxidant defense system), and endothelial tissue-1 (a potent vasoconstrictor) to inhibition of tissue macrophage infiltration in lung of MTX-intoxicated rats which affects the synthesis of interleukins. The present increase in both TNF-alpha and IL-6 confirms a pro-inflammatory role of these cytokines in MTX-induced toxicity.

It has been suggested that the action of MTX on pro-inflammatory cytokines is mediated by NF-kB. MTX injection, in the present study, showed strong positive expression of NF-kB in the inflamed lung sections. The activation of NF-kB can trigger cascades involved in the expression of iNOS and initiation of inflammation, cell proliferation, immune response and apoptosis^77^. It is activated by numerous stimuli as pro-inflammatory cytokines (TNF-alpha or IL-1β)^78^. Thus, the present increased expression of NF-kB may be mediated by the activation of TNF-α and IL-6 in the rat lung tissue by MTX injection.

The present results highlighted the inhibition of pro-inflammatory cytokines as one of the mechanisms by which PSO overcomes MTX toxicity.

Pumpkin oil was reported to contain polyphenols and other bioactive phytochemicals with anti-inflammatory effects^79^. Lai et al.^80^ indicated that the reduction of the inflammatory cellular infiltrate and the area percent of collagenous fibers after pumpkin oil administration in the lung of pumpkin oil and acid aspiration group were attributed to the unsaturated free fatty acid constituents in pumpkin oil.

Al-Okbi et al.^17^ attributed the bioactivity of PSO to linoleic acid which represented one-third of the total fatty acid. Omega-3 fatty acids can regulate the synthesis of lipid mediators, release of cytokines, and activate white blood cells and endothelial cells, thereby regulating the body’s excessive inflammatory response to reduce lung inflammation^47,81^.

Results in the present study concerning vascular endothelial growth factor (VEGF) level indicated a significant elevation of VEGF level in rat lung tissue of MTX-treated group.

The predominant source of VEGF in the lung is the alveolar epithelium, in addition to smooth muscle cells, macrophages, and endothelial cells which express VEGF. High levels of VEGF persist in the lungs in adulthood^82^ suggesting its role in normal lung maintenance and in the pathogenesis of acute respiratory distress syndrome (ARDS)^83^.

Thus, the increase in lung VEGF levels, in the present study, after MTX could be secondary to the increase in cytokine production as TNF-α and IL-6 have been reported to up regulate VEGF production in rheumatoid arthritis (RA)^84^.

It has been reported that the application of omega-3 fatty acids rapidly reduced the inflammatory reaction of lung tissue, by transforming leukotriene B4, which aggravates inflammatory reaction, into leukotriene B5 series (less active), therefore reducing pulmonary edema and improving pulmonary vascular permeability^85^. Huang et al.^86^ concluded that omega-3 acids can improve respiratory function and respiratory status of acute lung injury (ALI) patients and suggested that omega-3 fatty acid could be a potential effective and safe strategy for ALI treatment. The present restoration in VEGF levels after PSO treatment may be attributed to the potent anti-inflammatory properties of PSO constituents.

Apoptosis is a critical and vital process which occurs during chemically-induced toxicity. The present study showed a marked strong positive expression of the apoptotic marker caspase-3 in the interstitial tissue, alveoli and lining epithelium of bronchi and bronchioles of MTX group by immunohistochemical analysis. Caspase-3 is a key element in the apoptotic process and signals the induction of cell death^79^. Caspase-3 was considered as a valuable marker of apoptosis^87^. This may explain the severe lung injury detected in MTX-intoxicated rats.

The present findings are in agreement with the results of Kurt et al.^76^ who found similar histological damage in the MTX group with high caspase-3 expression. The authors attributed these changes to excessive cytokine levels, endothelial-1 secretion and ROS formation, which activate caspase-3 pathway causing lung damage. Crumbling of peptide chains, electric charge modification with cross-linking of proteins, and oxidation of specific amino acids, due to ROS and increased susceptibility to proteolysis by precise proteases^88^, may underlie the strong positive expression of caspase-3 and NF-κB detected in the immunohistochemical analysis in the present study in the lung interstitial tissue, alveoli and lining epithelium of bronchi and bronchioles of MTX group.

In the present study, PSO produced its protective effect by reducing inflammation and apoptosis, thereby attenuating and alleviating MTX-induced lung damage as evident in the histopathological examination which showed marked protection of the lung tissue with fewer numbers of inflammatory cells, mild edema and hemorrhage in the MTX + PSO group. Using the immunohistochemical technique, PSO was also shown to exert an antiapoptotic effect by reducing caspace 3 expression in the lung tissue of MTX-injected rats.

According to the results of the present study, it may be concluded that a single injection of MTX induces lung toxicity mediated by the increase in ROS, pro-inflammatory cytokines and transcription factors (NF-kappa). These alterations lead eventually to apoptosis and severe lung damage. A novel finding of the present study was the reduction of cytotoxicity and hence MTX-induced lung injury after PSO. The oral administration of PSO ameliorated most of the changes induced by MTX through the potent antioxidant, anti-inflammatory and anti-apoptotic activity of its constituents. The biochemical findings coincided with the histological examination indicating a marked protection of the lung tissue after PSO. The limitation of the present study was the use of a single dose of MTX and the short experimental period.

Thus, the pulmonary toxicity of MTX must be taken into consideration during its use in chemotherapy and in the treatment of RA and other diseases especially in patients suffering from pulmonary disorders. Patients under MTX therapy must also be monitored regularly to ensure that their lungs are not affected by MTX treatment. The use of natural antioxidants and anti-inflammatory agents is also highly recommended in patients under chemotherapy.

## Data Availability

All data generated or analyzed during this study are included in this published article. This study was carried out in accordance with ARRIVE guidelines.
